# Biomarkers in Rheumatoid Arthritis

**DOI:** 10.1155/2014/379310

**Published:** 2014-06-30

**Authors:** Vincent Goëb, Patrice Fardellone, Jean Sibilia, Frédérique Ponchel

**Affiliations:** ^1^Rheumatology Department, Research Units EA 4666 & Inserm U1088, Amiens University Hospital, University of Picardie Jules-Verne, 80054 Amiens, France; ^2^Rheumatology Department, Strasbourg University Hospital, Strasbourg, France; ^3^Leeds Institute of Rheumatic and Musculoskeletal Medicine, The University of Leeds, Leeds, UK

Rheumatoid arthritis (RA) is a chronic inflammatory autoimmune disease that leads to severe joint destruction with deformity and irremediable disability. Early diagnosis of RA and timely initiation of treatments (synthetic and/or biological disease modifying antirheumatic drugs) are both instrumental to limit joint damage and optimize the functional outcome of patients, according to the well-known concept of a “window of opportunity” to begin the treatments.

New diagnosis criteria are available since 2010, thanks to the American College of Rheumatology (ACR) and the European League Against Rheumatism (EULAR) [1], which include 2 different types of biomarkers: inflammation (CRP or ESR) and immunity (autoantibodies: rheumatoid factors (RF) and anticitrullinated peptides antibodies (ACPA)). These new criteria allow better management of patients with notably the early opportunity of treatment with methotrexate. However, a significant proportion of patients with early arthritis does not fulfil these criteria for RA and are then wrongly labelled “undifferentiated” arthritis [2]. Furthermore, among the autoantibodies family, ACPA are specific for the RA disease but lack sensitivity, unlike RF which have strong sensitivity but low specificity [3]. Thus there is still a need for new diagnosis biomarkers that would allow establishing a diagnosis of RA at the very beginning of the disease continuum, importantly before the occurrence of the first joint erosions.

Which type of biomarkers do we need? Several classes of markers are available: genetic polymorphisms, proteomic markers, gene-expression analysis [4], and autoantibodies. All may be used in clinical practice since their utility was demonstrated. In this special issue of “*Mediators of inflammation*,” novel biomarkers in RA are described. Patrice Fardellone et al. from the University of Picardy will discuss bone remodelling markers in RA, notably for bone formation (osteocalcin, serum aminoterminal propeptide of type I collagen) as well as bone resorption (C-terminal telopeptide of type 1 collagen, pyridinoline). They discuss how such bone remodelling markers allow physicians to evaluate the effect of drugs, notably biologicals that are able to reduce inflammation and also exert a protecting effect on bones. Furthermore, bone remodelling is the result of a tilted balance towards resorption or formation and involves numerous regulatory factors such as hormones, growth factors, vitamins, and cytokines, notably osteoprotegerin (OPG) and receptor activator for nuclear factor-*κ*B (RANK) ligand [5]. The signalling pathway OPG/RANK/RANKL maintains the balance between the activity of osteoblasts and osteoclasts. V. Milanova et al., from the Bulgarian Academy of Sciences, in “*TLR2 elicits IL-17-mediated RANKL expression, IL-17, and OPG production in neutrophils from arthritic mice,*” will show that the toll-like receptor 2 engagement increases IL-17 mediated RANKL expression and also inhibits OPG production in neutrophils from arthritic mice.

There is currently a great hope for biomarkers that would predict the response to treatment for individual patients in order to gain time and avoid irreversible damage, unnecessary risk of adverse events, as well as reduce long term disease associated cost, since uncontrolled inflammation over time leads to significant patient and health economic burden. I. Duroux-Richard et al. will thus explain how circulating micro-RNA, and notably miRNA-125b, are potential valuable biomarkers in RA in “*Circulating miRNA-125b is a potential biomarker predicting response to rituximab in rheumatoid arthritis*.” Circulating levels of miRNA-125 may predict the response to rituximab in RA patients and their interest must therefore be reassessed by other teams to be used in daily clinical practice.

Cytokine networks are well-recognized as a relevant source of contributive biomarkers in RA [6]. Adipokines are biological active substances synthesized by the white adipose tissue that regulate the energy homeostasis and metabolism and are soluble mediators involved in chronic inflammation and metabolic dysfunction. Their interest as biomarkers in RA is thus to be expected. Therefore, A. Burska et al. in “*Cytokines as biomarkers in rheumatoid arthritis*” and A. Del Prete et al. will explore the interests and limitations of using cytokines as biomarkers in RA with a special emphasis on adipokines for the latter.

Since the interest of autoantibodies in pathogenesis, diagnosis, and prognosis of different autoimmune diseases is obvious, the development of their knowledge is logical. Three main classes of posttranslational modifications are associated with RA: citrullination, of course, but also oxidation and carbamylation. A. Burska et al. will therefore review the relevance of autoantibodies to these 3 types of posttranslational modifications in RA in “*Cytokines as biomarkers in rheumatoid arthritis.*”

Remission with minimal use of drugs is now the goal of therapy for RA patients. Synovial (MMP3), cartilage (urinary CTX II, COMP), and bone biomarkers may be useful in managing drugs reduction when patients with RA achieved clinical remission. In this special issue, D. Dénarié et al. will try to shed light on the following issue: “*Could biomarkers of bone, cartilage, or synovium turnover be used for relapse prediction in RA patients?*” in this paper: “*Could biomarkers of bone, cartilage or synovium turnover be used for relapse prediction in rheumatoid arthritis patients?*”

We sincerely hope that this special issue of “*Mediators of inflammation on biomarkers in RA*” will be of interest to readers and deepen their knowledge of this subject. Kind regards.

*Vincent Goëb* *Vincent Goëb* *Patrice Fardellone* *Patrice Fardellone* *Jean Sibilia* *Jean Sibilia* *Frédérique Ponchel* *Frédérique Ponchel*
